# Genomic identification and characterization of *MYC* family genes in wheat (*Triticum aestivum* L.)

**DOI:** 10.1186/s12864-019-6373-y

**Published:** 2019-12-30

**Authors:** Jian-fang Bai, Yu-kun Wang, Li-ping Guo, Xiao-ming Guo, Hao-yu Guo, Shao-hua Yuan, Wen-jing Duan, Zihan Liu, Chang-ping Zhao, Feng-ting Zhang, Li-ping Zhang

**Affiliations:** 1 Beijing Engineering Research Center for Hybrid Wheat, Beijing Academy of Agriculture and Forestry Sciences, Beijing, 100097 China; 2 The Municipal Key Laboratory of Molecular Genetic of Hybrid Wheat, Beijing, 10097 China; 3 Division of Biological Science, Graduate School of Science and Technology, Nara Institute of Science and Technology, Nara, 630-0192 Japan; 4 School of Landscape Architecture, Beijing Forestry University, Beijing, 100083 People’s Republic of China

**Keywords:** Wheat, MYC, Gene family, JA signaling, Light response, Male sterile

## Abstract

**Background:**

*MYC* transcriptional factors are members of the *bHLH* (basic helix-loop-helix) superfamily, and play important roles in plant growth and development. Recent studies have revealed that some *MYCs* are involved in the crosstalk between Jasmonic acid regulatory pathway and light signaling in *Arabidopsis*, but such kinds of studies are rare in wheat, especially in photo-thermo-sensitive genic male sterile (PTGMS) wheat line.

**Results:**

27 non-redundant *MYC* gene copies, which belonged to 11 *TaMYC* genes, were identified in the whole genome of wheat (Chinese Spring). These gene copies were distributed on 13 different chromosomes, respectively. Based on the results of phylogenetic analysis, 27 *TaMYC* gene copies were clustered into group I, group III, and group IV. The identified *TaMYC* genes copies contained different numbers of light, stress, and hormone-responsive regulatory elements in their 1500 base pair promoter regions. Besides, we found that *TaMYC3* was expressed highly in stem, *TaMYC5* and *TaMYC9* were expressed specially in glume, and the rest of *TaMYC* genes were expressed in all tissues (root, stem, leaf, pistil, stamen, and glume) of the PTGMS line BS366. Moreover, we found that *TaMYC3, TaMYC7*, *TaMYC9*, and *TaMYC10* were highly sensitive to methyl jasmonate (MeJA), and other *TaMYC* genes responded at different levels. Furthermore, we confirmed the expression profiles of *TaMYC* family members under different light quality and plant hormone stimuli, and abiotic stresses. Finally, we predicted the wheat microRNAs that could interact with *TaMYC* family members, and built up a network to show their integrative relationships.

**Conclusions:**

This study analyzed the size and composition of the *MYC* gene family in wheat, and investigated stress-responsive and light quality induced expression profiles of each *TaMYC* gene in the PTGMS wheat line BS366. In conclusion, we obtained lots of important information of *TaMYC* family, and the results of this study was supposed to contribute novel insights and gene and microRNA resources for wheat breeding, especially for the improvement of PTGMS wheat lines.

## Background

The Jasmonic acid (JA) signaling pathway is complicated and is involved in several regulatory processes, such as plant growth and development, fertility regulation, and plant immunity [1, 2]. In *Arabidopsis*, components of JA signaling pathway include F-box protein CORONATINE INSENSITIVE1 (COI1), Jasmonate-ZIM (JAZ) domain repressor, and the bHLH transcription factor MYC2, which can regulate the expression patterns of JA-response genes [3]. JAZ proteins have been shown to block MYC2 activity in the absence of bioactive JAs by recruiting the general corepressors TOPLESS (TPL) and TPL-related proteins through the interaction with the adaptor protein novel interactor of JAZ (NINJA) [4]. In *Arabidopsis*, MYC2 was the first transcription factor (TF) found to be regulated by the JAZ proteins [3], and involved in defense regulation against insect herbivory via a partially redundant manner with its homologs MYC3 and MYC4 [5, 6]. These three MYC TFs can form homo- and heterodimers to bind G-box (CACGTG) elements or some variants of G-box elements [6]. However, these three MYC TFs play different roles in signaling pathways, despite they share the similar DNA binding sites. For instance, MYC3 and MYC4 are important for JA-mediated resistance to the herbivore *Spodoptora littoralis*, while the roles of MYC2, MYC3 and MYC4 are very weak in regulating JA-mediated inhibition of primary root growth. These differences might due to their preferential production in root and shoot tissues [5, 6]. In addition to the JA signaling pathway, MYC2 is also involved in repressing primary root growth, anthocyanin biosynthesis, oxidative stress tolerance [7], blue light-mediated photomorphogenic growth, resistance to necrotrophic fungi, and biosynthesis of tryptophan and indole-glucosinolates [8]. Therefore, MYC2 acts as a key factor that connects many pathways.

Among environmental signals, light is an important influential factor modulating plant growth and development. Light is a source of energy for plant photosynthesis and acts as a signal in the coordination of plant adaptive responses to environmental changes [9]. Plant responses to light are often mediated by photoreceptors, which are sensitive to specific wavelengths of the solar spectrum. Phytochromes belong an important class of photoreceptors play roles in light signaling. Phytochrome B (PHYB) is mainly sensitive to red light (R; wavelength: 660 nm) and PHYA is primarily sensitive to far-red light (FR; wavelength: 730 nm) radiation [10]. In *Arabidopsis,* under far-red light or darkness, plants show etiolation and elongated hypocotyls phenotypes, but the hypocotyl elongation is inhibited under red light conditions, which indicates that PHYA acts as a negative regulator of skotomorphogenesis, and PHYB is a positive regulator of photomorphogenesis [11]. Phytochromes proteins mainly include two types: the red light absorbing type (known as Pr) and the far-red light absorbing type (known as Pfr). They are interchangeable based on the R:FR ratios in environments [12]. Low R:FR ratio can reduce the levels of Pfr, and may be involved in shade-avoidance syndrome [11]. Recently, some studies have shown that components of JA signaling pathway, such as JAZ proteins, COIs and MYC2, are involved in several light-mediated responses. Robson et al. (2010) found that *Arabidopsis* mutations *jin1* and *myc2* are more sensitive to shade or FR light, and displayed an elongated hypocotyl phenotype under low R:FR ratio than wild type [13]. In addition, light-response related genes were upregulated under FR and blue light (BL) conditions in *jin1/myc2* mutant [14]. MYC2 could interact with the Z-box and G-box light-response elements and was thought as a negative regulator of blue light–mediated photomorphogenic growth [14]. Furthermore, MYC2 and SPA1 (suppressor of PHYA) may act redundantly in the dark and synergistically under light to suppress photomorphogenesis [15].

In addition, MYC2 also participates in the crosstalk among JAs and other plant hormones. In *Arabidopsis*, MYC2 was characterized as a transcriptional activator in ABA-inducible gene expression [16]. Song et al. (2014) demonstrated that MYC2 can interact with ethylene-stabilized transcription factor EIN3 and modulated JA and ET signal antagonism in *Arabidopsis* [17].

Wheat is one of the most important food crops. The fertility of the PTGMS wheat line BS366 is controlled by temperature and/or photoperiods [2]. The pollen of BS366 can not be fully spilled out, due to the impaired anther dehiscence, which can be recovered by the application of MeJA in vitro [2]. In wheat JA signaling pathway, 8 *COI* genes and 14 *JAZ* genes have been identified [2, 18]. However, few studies have been reported about wheat *MYC* gene family*,* which is an important component of the JA signaling pathway*.* In the present study, the *TaMYC* gene family was characterized using the latest genome sequences. We analyzed gene structures, conserved motifs, chromosome localization, and the regulatory networks of the *TaMYC* gene family. Given the important roles of microRNAs, such as miR1120 and miR1130 are involved in the JA signaling pathway and participate in anther development in wheat PTGMS line [19], we also predicted the interactive relationships between *TaMYC* and microRNAs. In addition, the expression profiles of *TaMYC* genes in the PTGMS wheat line BS366 were detected using qRT-PCR. Results in this study are expected to support a basis for further investigations on the functions of *TaMYC* genes, and provide some gene resources for revealing the molecular mechanisms of male sterility in PTGMS wheat.

## Results

### Identification of *TaMYC* gene family

After the removal of redundant gene, 27 non-redundant *MYC* gene copies, which belonged to 11 *MYC* genes, were identified. Firstly, we monitored the physical and chemical characteristics of these *MYC* gene copies. The coding sequence lengths of 27 *MYC* gene copies were ranged from 1332 bp to 2088 bp, and the deduced protein lengths were ranged from 443 to 695 amino acids (Table 1). The predicted molecular weights (MWs) of each MYC protein were ranged from 47.53 kDa to 75.62 kDa, and the corresponding isoelectric points (IPs) were changed from 4.96 to 8.73 (Table 1). Subcellular localization predictions revealed that MYC proteins were functioned in chloroplast, cytoplasmic, nuclear, or plasma membrane (Table 1). Different characteristics of *TaMYC* genes and proteins were obtained, and the results indicated that different TaMYC proteins might have different biological functions.

**Table 1:** Characteristics of *TaMYC* gene family members

Gene name	Sequence ID	Locations	Protein/AA	Isoelectric point	Molecular weight of decuced protein/KD	Subcellular Localization
*TaMYC1-A*	TraesCS1A02G102400	1A:98584808:98591571:+	626	5.65	68.48	Nuclear
*TaMYC2-A*	TraesCS1A02G193200	1A:349358465:349360851:+	693	6.37	74.04	Nuclear
*TaMYC1-B1*	TraesCS1B02G112900	1B:131456949:131464291:+	631	5.84	69.22	Nuclear
*TaMYC1-B2*	TraesCS1B02G113100	1B:131775632:131785030:+	688	5.87	75.62	Nuclear
*TaMYC2-B*	TraesCS1B02G208000	1B:376071131:376073212:+	693	6.15	75.09	Nuclear
*TaMYC2-D*	TraesCS1D02G196900	1D:277092891:277094978:+	695	6.15	75.35	Nuclear
*TaMYC3-A1*	TraesCS2A02G409400	2A:667011017:667015600:+	568	5.37	62.65	Chloroplast|Cytoplasmic
*TaMYC3-A2*	TraesCS2A02G409600	2A:667647129:667652609:+	558	5.54	60.82	Chloroplast|Cytoplasmic
*TaMYC3-B*	TraesCS2B02G428000	2B:615352721:615356928:+	573	5.17	62.77	Chloroplast
*TaMYC7-D*	TraesCS2D02G406800	2D:522323574:522329854:+	465	4.96	51.53	Cytoplasmic
*TaMYC3-D*	TraesCS2D02G406900	2D:522521895:522526799:+	567	5.19	61.82	Cytoplasmic|Chloroplast
*TaMYC8-D*	TraesCS2D02G575600	2D:639529624:639535060:-	589	5.24	66.17	Cytoplasmic
*TaMYC4-A*	TraesCS3A02G158600	3A:156267999:156270204:-	616	6.52	67.35	Nuclear|Cytoplasmic
*TaMYC5-A*	TraesCS3A02G252900	3A:474209127:474210506:-	459	8.43	49.17	Cytoplasmic|Mitochondrial
Gene name	Sequence ID	Locations	Protein/AA	Isoelectric point	Molecular weight of decuced protein/KD	Subcellular Localization
*TaMYC4-B*	TraesCS3B02G185400	3B:201339407:201341777:-	625	7.11	68.24	Nuclear|Cytoplasmic
*TaMYC5-B1*	TraesCS3B02G288700	3B:463203072:463205129:+	443	8.47	47.53	Cytoplasmic|Mitochondrial
*TaMYC5-B2*	TraesCS3B02G284800	3B:456323130:456324920:-	475	7.77	50.76	Cytoplasmic|Mitochondrial
*TaMYC4-D*	TraesCS3D02G166300	3D:138192597:138194964:-	625	6.76	68.31	Nuclear|Cytoplasmic
*TaMYC5-D*	TraesCS3D02G253700	3D:355318384:355320162:-	465	8.73	49.82	Mitochondrial|Cytoplasmic
*TaMYC6-A*	TraesCS4A02G028900	4A:21063512:21065305:-	597	6.56	65.55	Nuclear|Cytoplasmic
*TaMYC6-B*	TraesCS4B02G276900	4B:558578472:558580268:+	598	6.73	65.56	Nuclear|Cytoplasmic
*TaMYC8-B*	TraesCS4B02G397400	4B:671703616:671707933:+	564	5.18	62.86	Chloroplast|Cytoplasmic
*TaMYC9-B*	TraesCS4B02G397800	4B:671764988:671776128:-	510	5.47	57.00	Plasma Membrane|Nuclear
*TaMYC10-D*	TraesCS4D02G224600	4D:381551243:381559778:+	557	6.49	61.40	Mitochondrial|Cytoplasmic
*TaMYC6-D*	TraesCS4D02G275500	4D:446428380:446430176:+	598	6.56	65.59	Nuclear|Cytoplasmic
*TaMYC11-A1*	TraesCS5A02G489500	5A:659338425:659342323:-	570	5.31	63.15	Cytoplasmic|Mitochondrial
*TaMYC11-A2*	TraesCS5A02G558500	5A:709193171:709197273:+	585	5.26	64.93	Cytoplasmic

### Analysis of chromosomal locations and synteny

**Fig. 1:**
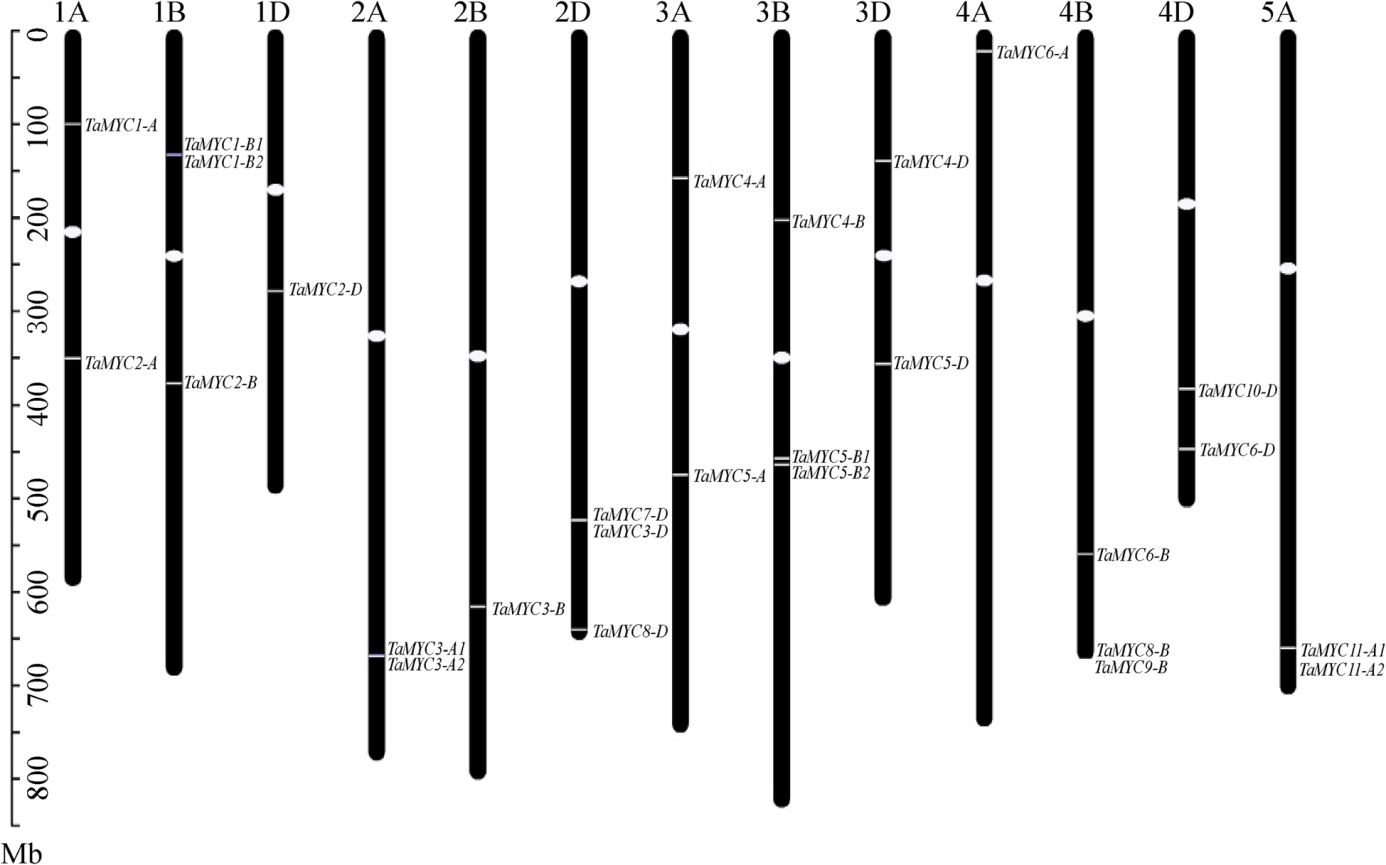
Chromosomal distribution of *TaMYC* gene copies. Only those chromosomes containing *TaMYC* genes are represented

In order to understand the relative position of each *TaMYC* gene copy on wheat chromosomes, we marked their physical placements on wheat A, B, and D chromosomes. As shown in Figs. 1, 27 *TaMYC* gene copies were located on 13 chromosomes. 9, 10, and 8 *TaMYC* gene copies were located on chromosomes 1A-5A, 1B-4B, and 1D-4D, respectively (Fig. 1).

Gene and chromosomal segment duplication are the major forces of genome evolution in plants [20]. In the wheat genome, four tandem duplication events (*TaMYC1-B1/TaMYC1-B2*, *TaMYC3-A1/TaMYC3-A2*, *TaMYC5-B1/TaMYC5-B2,* and *TaMYC11A-1/TaMYC11A-2*) and 25 segmental duplication events were identified, which indicate that segmental duplication events were the main cause of the increase of *MYC* members in wheat (Additional file 1: Figure S1and Additional file 7: Table S4). Synteny analysis among *TaMYCs* and its ancestors were also analyzed. Nine members (*TaMYC1-A*, *TaMYC3-A2*, *TaMYC4-A*, *TaMYC6-A*, *TaMYC2-D*, *TaMYC3-D*, *TaMYC4-D*, *TaMYC5-D* and *TaMYC6-D*) of the *TaMYC* gene family have homology with genes of *T. urartu* and *Ae. tauschii* (Additional file 1: Fig. S1)*.*

### Phylogenetic analysis of TaMYC proteins

**Fig. 2:**
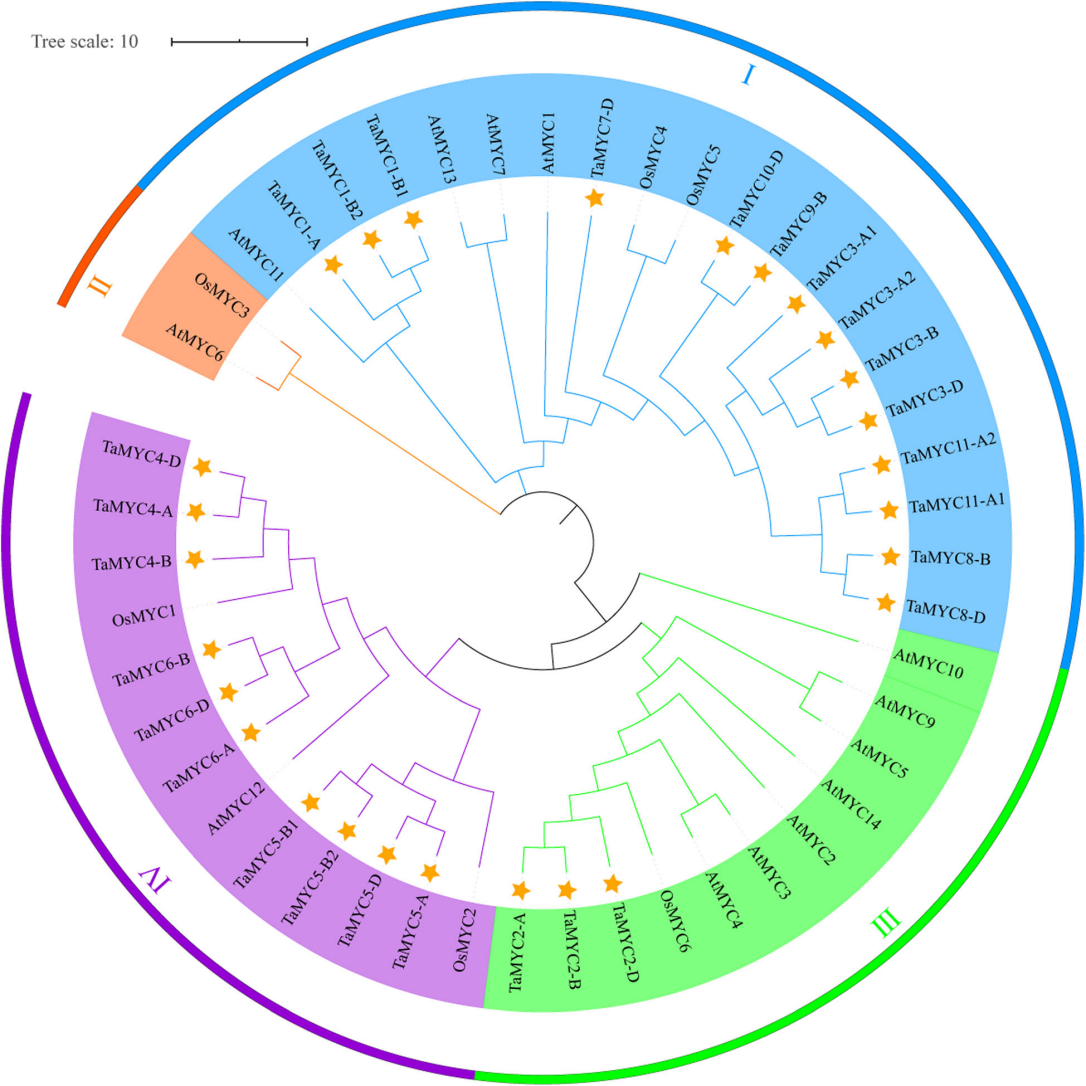
Unrooted phylogenetic tree based on protein alignments. MYC proteins from wheat (Ta), *Arabidopsis thaliana* (At), and rice (Os) were used. The neighbor-joining method is used with 1000 bootstrap trials. The yellow pentagrams indicated TaMYC proteins

To reveal the functional information of *TaMYC* genes, a phylogenetic tree, which based on the compare among wheat, *Arabidopsis*, and rice, was constructed using N-J method. As shown in Fig. 2, MYC proteins of three species were clustered into four groups (I, II, III, and IV). TaMYCs were distributed in groups I, III and IV. Seven TaMYC proteins (16 copies) were clustered into group I, while only TaMYC2 (three copies) was clustered into Group III. It was worth noting that three copies of TaMYC2 had a close homology with AtMYC2, AtMYC3 and AtMYC4, which are genes that have been demonstrated to play similar roles in plant development (Fig. 2).

### Structural analysis of *TaMYC* genes and proteins

**Fig. 3:**
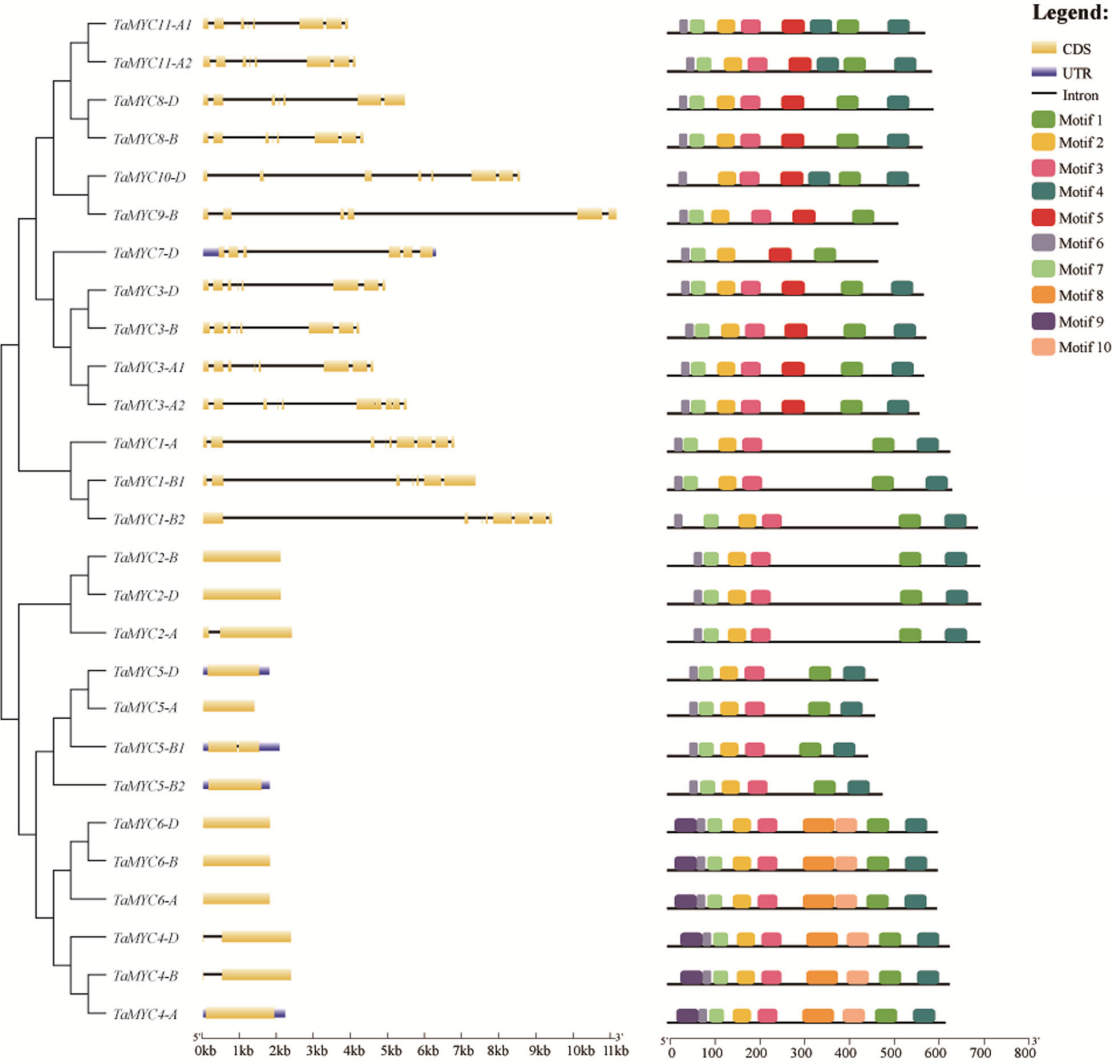
Exon-intron structures and motif distribution of *TaMYC* genes and TaMYC proteins. Exons are shown as yellow boxes, introns are shown as thin lines, and UTRs are shown as blue boxes. The sizes of exons and introns can be estimated using the scale below. Ten motif types are shown as colored boxes listed at right side

For understanding the structural features of *TaMYC* family members, firstly, the exon-intron structural features were revealed by aligning the predicted CDS against corresponding genomic sequences. As shown in Fig. 3, the intron–exon structures of different *TaMYC* genes were diverse, while copies of the same genes were similar or same, such as *TaMYC6* (*TaMYC6-A*, *TaMYC6-B* and *TaMYC6-D*) and *TaMYC11 (TaMYC11-A1 and TaMYC11-A2)*. It was notable that *MYC* genes of the same subgroup shared similar intron–exon structures, for instance, *TaMYC4* and *TaMYC6* were both in subgroup IV, and they only had one exon (Fig.2 and Fig. 3).

Motifs in TaMYC proteins were also predicted in this study. Similar with the intron–exon structures of *TaMYC* genes, proteins of the same subgroup shared the same or similar motifs. Most copies of TaMYC proteins possessed six, seven or eight motifs (Fig. 3). TaMYC7-D only had five motifs (Fig. 3). As the member of bHLH superfamily, MYC family proteins contain a bHLH domain and a conserved bHLH-MYC_N domain [15, 21]. In this study, motifs 2, 3, 6 and 7 corresponded to bHLH-MYC_N domains, while motifs 1 and 4 corresponded to bHLH domains (Additional file 2: Fig. S2). The biological functions of other motifs remains unknown, but it could be predicted that some TaMYC proteins had unknown functions.

### Analysis of *cis*-regulatory elements of *TaMYC* genes

**Fig. 4:**
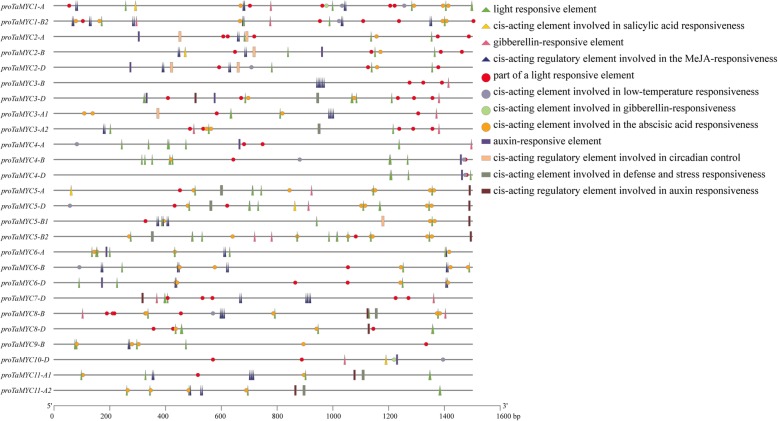
The number and composition of *cis*-acting regulatory elements in the promotor region of *TaMYC* genes. 1500 base pair promoter region of each gene copy is displayed. Different colorful shapes show different elements

The upstream promoter regions (1500 bp, Additional file 3: File S1) of *TaMYC* gene copies were retrieved from the wheat genome to identify *cis*-regulatory elements. Five light-responsiveness regulatory elements, including ACE, ATC-motif, Box 4, G-box and MRE were identified in all TaMYC promoter regions, indicating that *TaMYC*s might be involved in light signaling pathways (Fig. 4 and Additional file 5: Table S2). Eight hormone-responsive regulatory elements, including TGA-element, TCA-element, TATC-box, P-box, GARE-motif, CGTCA/TGACG-motif, AuxRR-core, and ABRE, which were associated with auxin, salicylic acid, gibberellin, MeJA, and ABA responses, were identified in the promoter region of *TaMYC* copies (Fig. 4 and Additional file 5: Table S2). Besides, six stress-responsive regulatory elements, WUN-motif, TC-rich repeats, MBS, LTR, GC-motif and ARE, which were associated with wound responsiveness, defense and stress responsiveness, drought-inducibility, low-temperature responsiveness, anoxic specific inducibility and anaerobic induction, respectively, were identified (Fig. 4 and Additional file 5: Table S2). In addition, *cis*-regulatory elements for five regulators of development regulation and two regulators of biosynthesis regulation were identified. As shown in Additional file 5: Table S2, different types and numbers of regulatory elements were identified in the promoter regions of different *TaMYC* genes, indicating that *TaMYC* genes might have different functions in stress resistance, growth and development.

### Tissue/organ-specific expression profiles of *TaMYC* genes

**Fig. 5:**
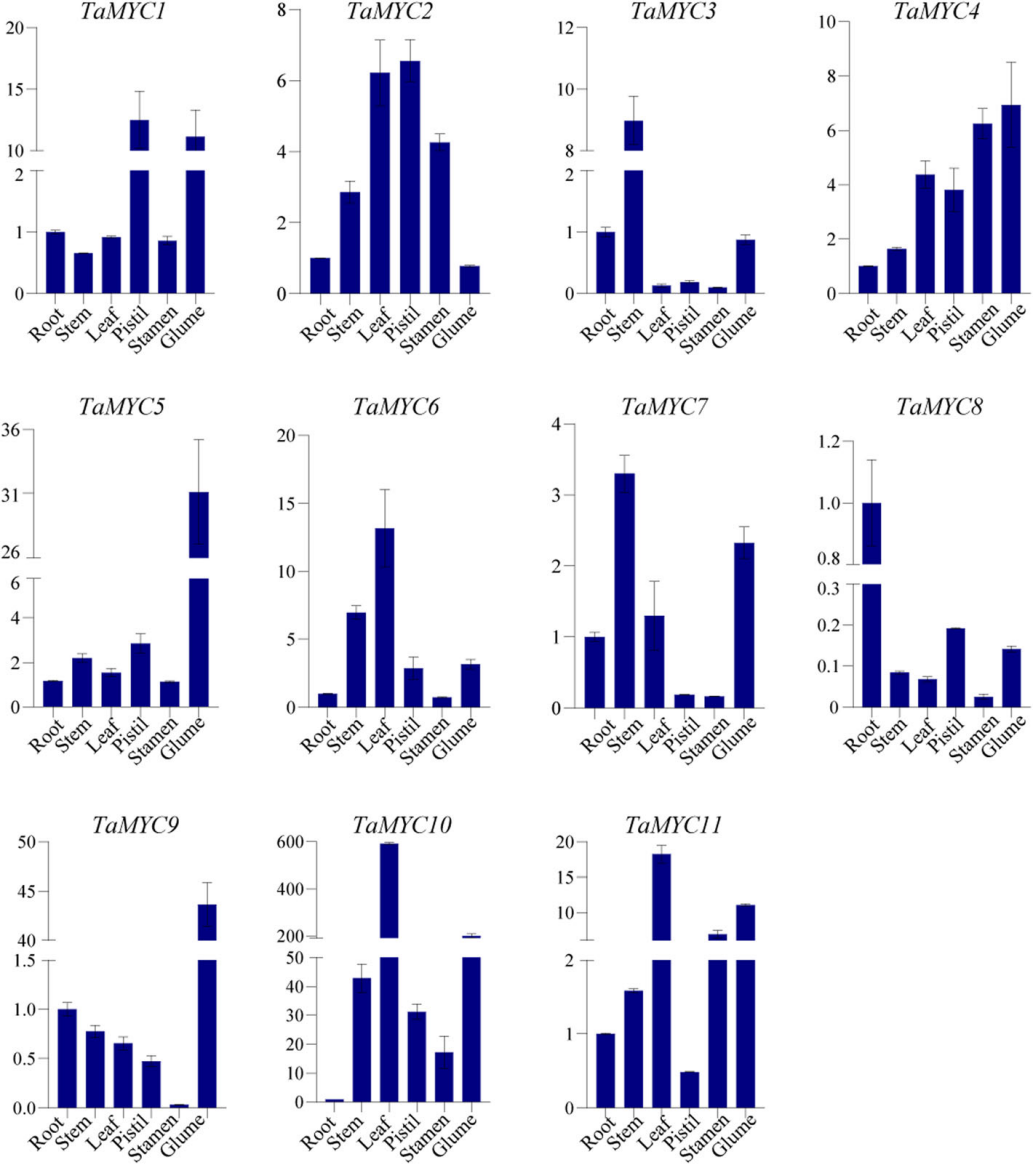
Relative expression of *TaMYC* genes in six wheat tissues (root, stem, leaf, pistil, stamen, and glume) in the heading stage by qRT-PCR. The error bars indicate the standard deviation obtained from three replications. Wheat actin gene is used as the inner reference

To study the tissue/organ-specific expression patterns of the 11 identified *TaMYC* genes, their expression patterns in root, stem, leaf, petal, pistil, stamen, and glume of the PTGMS wheat line BS366 were investigated by qRT-PCR. As shown in Figs. 5, 11 *TaMYC* genes showed different expression levels in different tissues. The expression levels of *TaMYC5* and *TaMYC9* in glume were higher than that in other tissues (Fig. 5). In addition, *TaMYC1* and *TaMYC2* had relatively high expression levels in pistil tissue, while *TaMYC6*, *TaMYC10*, and *TaMYC11* had relatively high expression in leaf tissue (Fig. 5). *TaMYC4* was constitutively expressed in all six tissues (Fig. 5). Meanwhile, we noticed that *TaMYC2*, *TaMYC4*, *TaMYC10*, and *TaMYC11* displayed relatively high expression levels in stamen (Fig. 5).

### Effects of exogenetic MeJA treatment

**Fig. 6:**
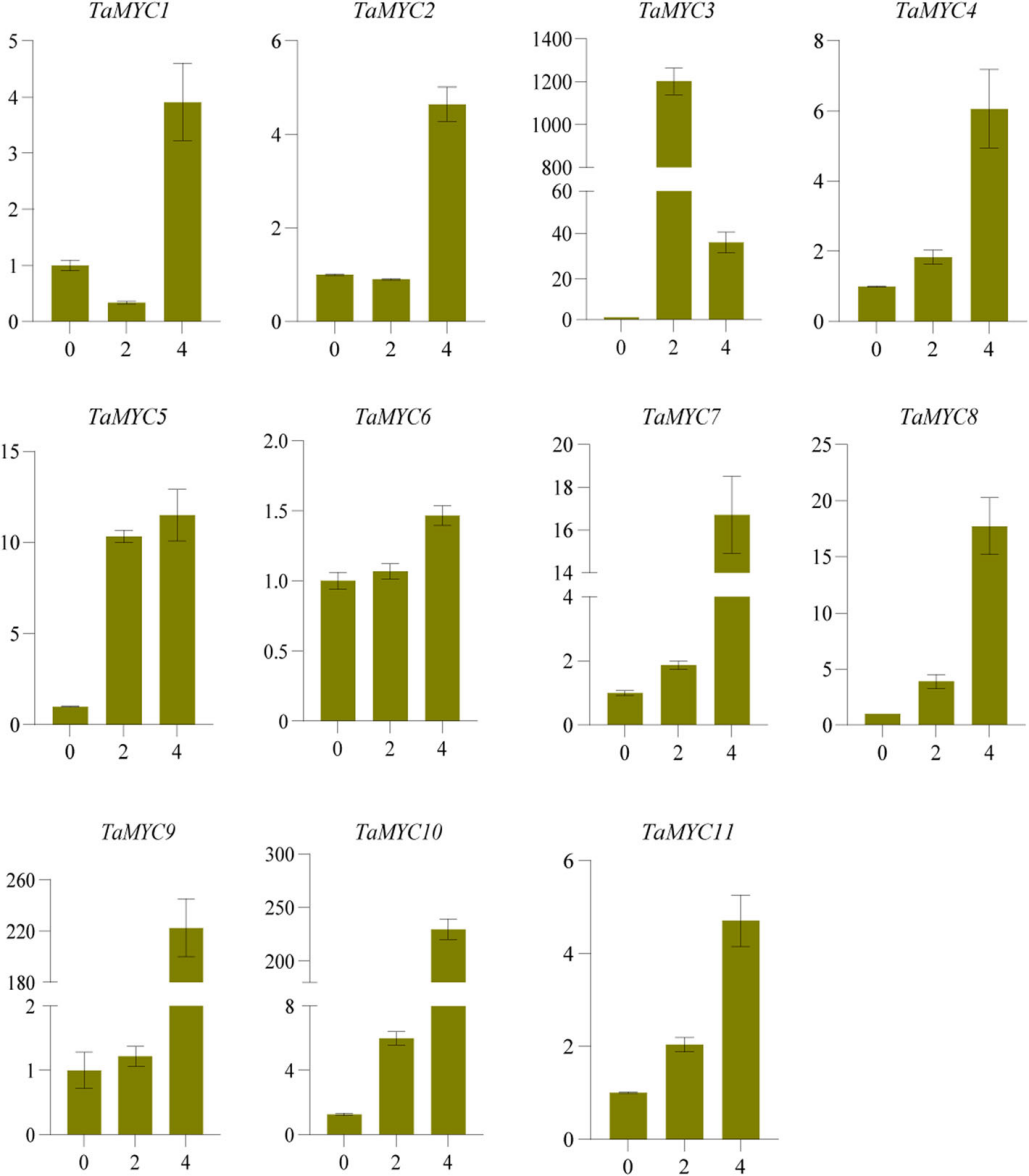
Relative expression of *TaMYC* genes in anthers treated with different concentrations of MeJA. Values on the x-axis indicate the MeJA concentration. MeJA: spikelets were treated with MeJA (0 mM, 2 mM, and 4 mM). The error bars indicate the standard deviation obtained from three replications. Wheat *Actin* gene is used as the inner reference

In order to investigate the functions of *MYCs* in the JA signaling pathway at anther dehiscent stage, the expression profiles of *TaMYC* genes in anthers, which treated with different concentrations of MeJA, were analyzed. As shown in Fig. 6, the expression of *TaMYC3* was high under 2 mmol/L MeJA. The expression levels of T*aMYC5*, *TaMYC7*, *TaMYC8*, *TaMYC9*, and *TaMYC10* in anther were induced by 4 mmol/L MeJA (Fig. 6). These results indicate that these *TaMYC* genes could be strongly induced by MeJA and might be function on the JA signaling pathway in anther of the PTGMS wheat line BS366.

### Photochromic conversion-induced expression profiles of *TaMYC* genes

**Fig. 7:**
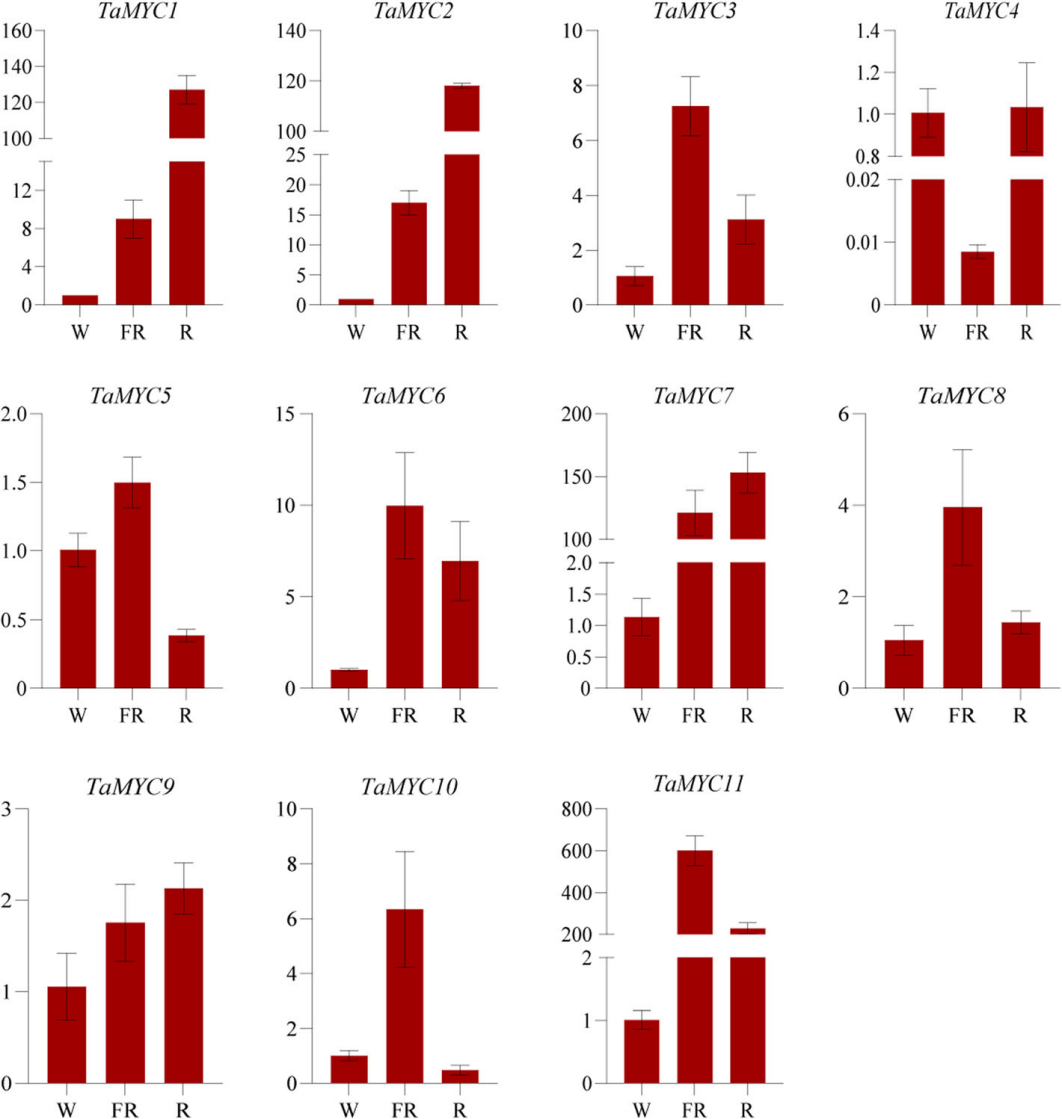
Relative expression of *TaMYC* genes in anthers treated with different lights. The error bars indicate the standard deviation obtained from three replications. Wheat *Actin* gene is used as the inner reference

Many studies have suggested that MYC may be involved in the cross-talk between the JA signaling pathway and light signaling pathway (R:FR ratio mediated Pfr/Pr conversion) [12]. To investigate photochromic conversion-induced expression profiles of *TaMYC* genes, different R: FR ration light treatments were performed on BS366 seedlings. As shown in Fig. 7, *TaMYC1*, *TaMYC2*, *TaMYC6*, *TaMYC7*, *TaMYC8*, *TaMYC9* and *TaMYC11* were upregulated in R and FR light-enriched conditions compared to white light conditions. In addition, *TaMYC4* was downregulated by far-red light, and *TaMYC5* and *TaMYC10* were inhibited under R light condition (Fig. 7).

### Expression profiles of *TaMYC* genes under phytohormone treatments

**Fig. 8:**
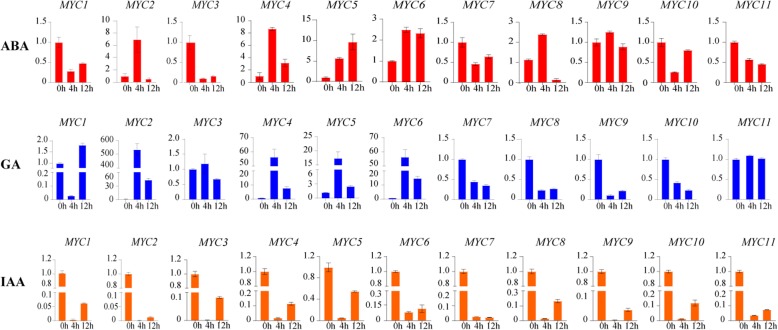
Relative expression of *TaMYC* genes under phytohormone treatments. The error bars indicate the standard deviation obtained from three replications. Wheat actin gene is used as the inner reference

The expression profiles of *TaMYC* genes in the leaf tissues of the PTGMS line BS366 under plant hormones treatments were analyzed to determine the responsive profiles. Under ABA treatment, the expression levels of *TaMYC2, TaMYC8,* and *TaMYC9* were upregulated at 4 h post-treatment, then downregulated at 12 h post-treatment (Fig. 8). *TaMYC4*, *TaMYC5* and *TaMYC6* were induced after ABA treatment. *TaMYC1*, *TaMYC3*, *TaMYC7*, *TaMYC10* and *TaMYC11* showed negative responses to ABA treatment (Fig. 8). Under GA treatment, the expression of *TaMYC7*, *TaMYC8*, *TaMYC9*, and *TaMYC10* were inhibited, while the expression of *TaMYC2*, *TaMYC4*, *TaMYC5*, and *TaMYC6* were promoted (Fig. 8). The transcript profiles of *TaMYC3* and *TaMYC11* were slightly upregulated and peaked at 4 h post-treatment. *TaMYC1* was downregulated at 4 h post-treatment, but showed an increase profile at 12 h post-treatment (Fig. 8). Under IAA treatment, the expressions of all *TaMYC* genes were downregulated (Fig. 8).

### Abiotic stress-induced expression profiles of *TaMYC* genes

**Fig. 9:**
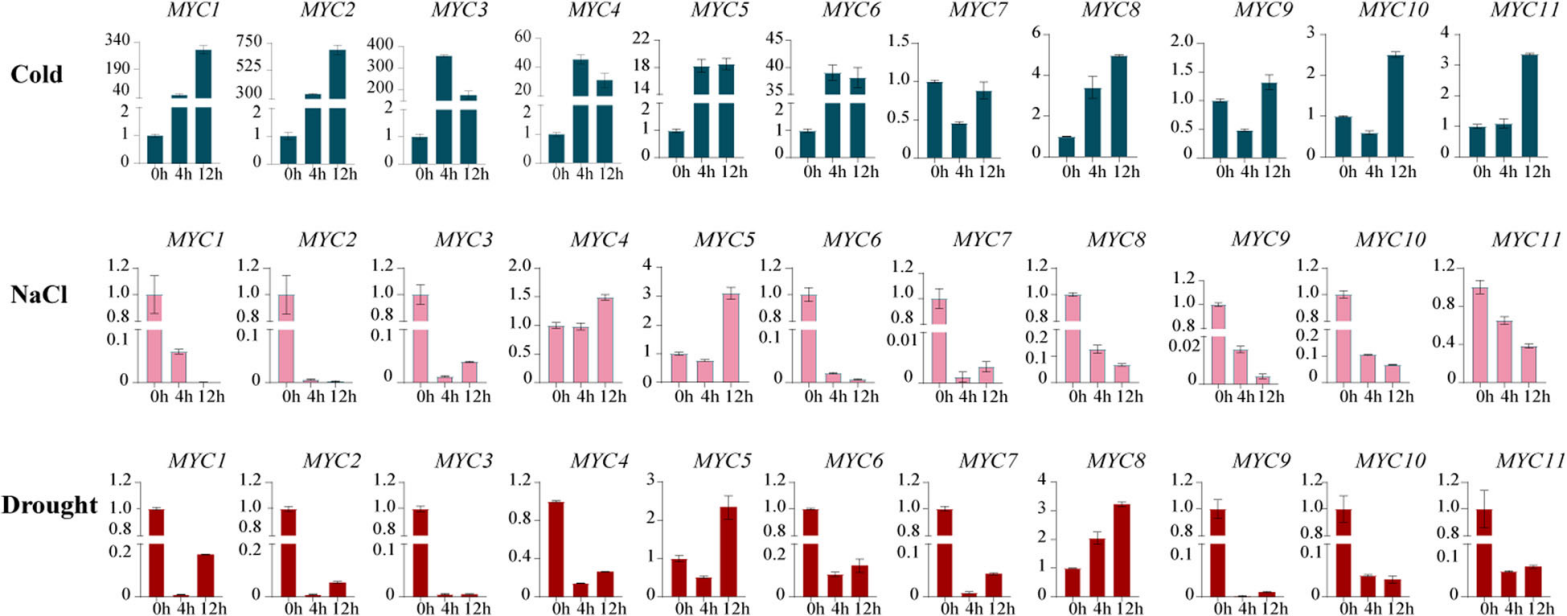
Relative expression of *TaMYC* genes under abiotic stresses. The error bars indicate the standard deviation obtained from three replications. Wheat actin gene is used as the inner reference

The transcriptional profiles of *TaMYC* genes under abiotic stresses were monitored in this study. As shown in Fig. 9, the expressions of *TaMYC1*, *TaMYC2*, *TaMYC3*, *TaMYC4*, *TaMYC5*, *TaMYC6, TaMYC8*, *TaMYC10*, and *TaMYC11* were upregulated under low temperature treatment. The expression levels of *TaMYC7* and *TaMYC9* were decreased at 4 h post-treatment, and recovered at 12 h post-treatment (Fig. 9). Under high salinity conditions, *TaMYC1*, *TaMYC2*, *TaMYC3*, *TaMYC6*, *TaMYC7*, *TaMYC8*, *TaMYC9*, *TaMYC10*, and *TaMYC11* were downregulated. Only *TaMYC4* and *TaMYC5* were promoted after saline treatment (Fig. 9). Under drought stress, *TaMYC5* and *TaMYC8* were upregulated, while the rest of *TaMYCs* were downregulated (Fig. 9).

### microRNA targeting prediction of *TaMYC* genes

**Fig. 10:**
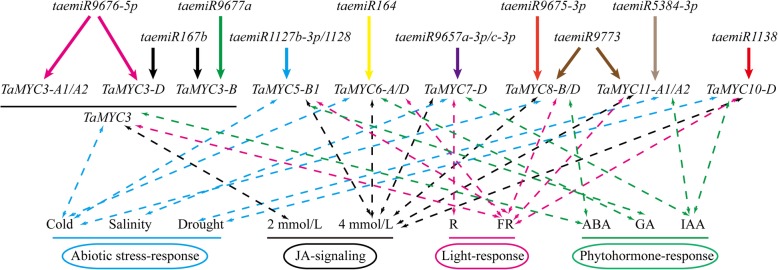
A putative network of wheat miRNAs and targeted *TaMYC* genes. The putative binding sequence of each pair of miRNA and target is listed in Additional file 6: Table S3. The bidirectional and dotted arrow lines show the most sensitive responses to stresses of each *MYC* gene. The unidirectional arrow lines indicate the interactions between taemiRNAs and target *MYC* genes

In order to uncover the interactions between microRNAs (miRNAs) and their *MYC* targets, we predicted the potential networks using online tool (Additional file 6: Table S3). As shown in Fig. 10, seven *TaMYC* genes, including *TaMYC3*, *TaMYC5*, *TaMYC6*, *TaMYC7*, *TaMYC8*, *TaMYC10*, *TaMYC11*, were regulated by 12 miRNAs (taemiR1127b-3p, taemiR9657a-3p, taemiR9676-5p, taemiR1138, taemiR167b, taemiR5384-3p, taemiR9773, taemiR1128, taemiR164, taemiR9657c-3p, taemiR9675-3p, and taemiR9677a). We speculated these miRNAs could inhibit the expression levels of target *TaMYCs* at the transcriptional or translational level. One *TaMYC* gene might be targeted by multiple miRNAs, while several *TaMYC* genes might be regulated by the same miRNA (Fig. 10). In order to understand the potential functions, we pointed out the response model of each target *TaMYC* gene under different stresses or treatments. *TaMYC3*, *TaMYC5*, *TaMYC6*, and *TaMYC8* were responded to cold stress, *TaMYC7* was mainly responded to salinity, and *TaMYC10* and *TaMYC11* were thought to show a close relationship with drought stress (Fig. 10). Only *TaMYC3* showed a clear response to 2 mmol/L MeJA treatment, and the rest of 6 *TaMYCs* were promoted at 4 mmol/L MeJA treatment (Fig. 10). *TaMYC7-D* was sensitive to red light stimulus, while others were more sensitive to far red light (Fig. 10). Besides, we found that *TaMYC3* and *TaMYC8* were regulated by ABA, *TaMYC5* and *TaMYC6* could response to GA, and *TaMYC7*, *TaMYC10*, and *TaMYC11* were mainly regulated by IAA (Fig. 10). Although the precise functions of most miRNAs in this study were unknown, some miRNAs (such as taemiR167 and taemiR1127b-3p) had already identified as factors involved in the regulation of male sterility in wheat [2, 19]. Based on the network shown in Fig. 10, we thought that *TaMYC3*, *TaMYC5*, *TaMYC6*, and *TaMYC8* maintained high possibilities on the regulation of temperature-induced male sterility in wheat PTGMS line BS366.

## Discussion

The JA signaling pathway is involved in plant growth and development, and it can regulate the plant fertility [22], anthocyanin accumulation [23] and leaf senescence [24]. MYC is an important component of the COI1/JAZ/MYC2 complex and can mediate JA and various hormone signaling pathways, and the light signaling pathway [12]. However, there are few reports regarding the function of MYC in crop plants. In the present study, aiming to understand the roles of *MYC* genes in wheat, 27 candidate *TaMYC* copies were isolated (Fig. 1 and Table 1) and clustered into 11 *MYC* genes (Fig. 2). These *TaMYC* genes were classified into 3 subgroups based on phylogenetic analysis and shared relatively close homology with MYCs of *Arabidopsi* and rice (Fig. 2). Furthermore, phylogenetic analysis revealed that *TaMYC2* had close homology with *AtMYC2*, *AtMYC3* and *AtMYC4,* indicating that *TaMYC2* may have similar roles in regulating wheat development (Fig. 2).

*Cis*-regulatory sequences, such as enhancers and promoters, can control development and physiology by regulating gene expression [25]. *Cis*-regulatory elements, acting as important molecular switches, are involved in the regulation of gene transcription under external stimuli [26]. To clarify the functions of *TaMYC* genes, *cis*-regulatory elements in the promoter regions of *TaMYC* genes, and their expression profiles under various stresses were analyzed. It was found that six stress-responsive elements, five light-responsive elements, and eight hormone-responsive elements were identified in *TaMYC* promoter regions (Fig. 4 and Additional file 6: Table S3). The 11 *TaMYC*s contained different types and numbers of *cis*-acting regulatory elements in each promoter region, indicating that these genes had different regulatory functions that responded to different stress and hormone treatments. Furthermore, we evaluated the expression profiles of *TaMYC* genes in PTGMS wheat line BS366 seedlings under different stress conditions and hormone treatments. Inducible expression analyses revealed that both *TaMYC4* and *TaMYC6,* with similar protein structures (Fig. 3), were up-regulated by treatments of ABA, GA, low temperatures, and drought (Fig. 8 and Fig.9). Similar expression profile trends also exited in *TaMYC1* and *TaMYC2* under treatment with low temperature and salinity treatments (Fig. 9).

Phytohormone crosstalk involves a very complex signaling regulative network, is universally available in plants, and plays a very important role in plant regulation of growth and development as the role of in JA signaling pathway [27]. *TaMYC* genes were found to contain different numbers of hormone-responsive elements, especially ABA (ABRE) and MeJA (CGTCA/TGACG-motif) (Additional file 5: Table S2). ABA is a “stress hormone” that can regulate plant growth, stress tolerance, seed germination, and tissue/organ senescence [18]. In *Arabidopsis*, *MYC2* functions in ABA signaling pathways [16]. In this study, *TaMYC2, 4, 5, 6,* and *8* were upregulated under ABA treatment (Fig. 8). It is known that MeJA may play a positive regulatory role in anther dehiscence and glume opening [2, 28]. As an important component of the JA signaling pathway, *TaMYCs* were also regulated by MeJA. For example, the promoter region of *TaMYC3* was rich in CGTCA/TGACG-motifs (numbers of motifs were 2, 1, and 3 for *TaMYC3a-A, TaMYC3b-A, and TaMYC3-B*, respectively) (Fig. 4 and Additional file 5: Table S2), and they displayed very high expression levels after MeJA treatment (Fig. 6). Meanwhile, the transcriptional levels of the rest of *TaMYC* were significantly induced by different concentrations of MeJA (Fig. 6), although there were no CGTCA/TGACG-motifs in the promoter region of *TaMYC4, 5 and 10*. This indicated that the gene expression level under different treatments was not only dependent on the presence of relevant *cis*-acting regulatory elements, but also might be regulated by other physiological pathways in wheat.

A growing body of evidences indicate that the JA response is modulated by the ecological context of the plant, especially light, which is emerging as a critical regulator of JA signaling [29]. In *Arabidopsis*, MYC2 can interact with SPA1 (suppressor of PHYA) redundantly in the dark and synergistically in the light to suppress photomorphogenesis by binding to the G-box (found in the promoter of SPA1) [21]. In this study, we also found that most of *TaMYC* genes were rich in G-box *cis*-acting regulatory elements. For example, *TaMYC1*, *TaMYC5*, *TaMYC6*, and *TaMYC11* contained at least 4 G-box motifs, which are involved in light responsiveness (Fig. 4 and Additional file 5: Table S2). The state of conversion between Pfr and Pr is involved in detecting the quality of light by monitoring R:FR ratios [30]. Some evidences show that FR light-enriched environments could inactivate PHYB and promote the shade avoidance syndrome [30]. To investigate the possible functions of *TaMYCs* in light signaling, we utilized the R: FR ratio-based Pfr/Pr conversion experiment. The results showed that all *TaMYC*s were induced by R and FR, such as the expression of *TaMYC1, 2, 3, 6, 7, 8, 9* and *11*, which were up-regulated under both low and high R:FR ratios in the dark (Fig. 7). Here, the expression of *TaMYC4* was down-regulated under low R:FR ratios (far-red light-enriched environment), and expression of *TaMYC5* was down-regulated under high R:FR ratios (red light-enriched environment), indicating that these two genes might be regulated by other crosstalk pathways in wheat line BS366. In *coi1* mutants, the expressions of JA biosynthetic gene (e.g. *AOC1*), signaling gene (*JAZ1* and *MYC2*), and wound response (*VSP1*) genes were attenuated, suggesting that FR light is a positive regulator of JA-responsive gene expression [13]. In this study, *TaMYC* genes showed different expression patterns under FR or R light-enriched environments, indicating they might differentially regulate different branches of the JA signaling pathway.

Many recent studies have indicated that miRNAs are involved in anther development and male sterility [19]. To further understand the roles of *TaMYCs*, the interaction relationship between *TaMYCs* and wheat miRNAs were predicted. Totally, seven *TaMYCs* were targeted by 12 taemiRNAs, including taemiR164 and taemiR167, which play important roles in plant growth and development (Fig. 10). In plants, miR164 targeted genes participate in various physiological and biochemical processes during plant development and in response to biotic/abiotic stress [31, 32]. For example, miR164 is involved in age-dependent cell death in *Arabidopsis* leaves by cleaving ORE (A *NAM* transcription factor) [33]. Plants with mutated *miR164-CUC1* and *miR164-CUC2* exhibit multiple phenotypes, such as leaf shape and polarity defects, extra petals, missing sepals, and reduced fertility [34, 35]. In addition, miR164 can also be regulated by light, and its expression was up-regulated under UV-B radiation treatment in maize [36]. In the present study, *TaMYC6* responded to red/far-red light stimulation, hormone treatment, low temperatures stress, and drought stress, and might be targeted by miR164 (Fig. 10). In *Arabidopsis*, miR167 targeting Auxin Response Factor 6 (*ARF6*), and *ARF8*, which regulates jasmonate biosynthesis by inhibiting downstream genes, regulates pollen development [37]. Wang et al. (2019) demonstrate that taemiR167, can induce male sterility and reduce the expression levels of *AtARF6* and *AtARF8* as well as reduce the content of JA and IAA in transgenic *Arabidopsis* [38]. One study shows that miR167 expression is upregulated in the light and downregulated in darkness, suggesting that miR167 may be regulated by light or the circadian clock [39]. Here, the results indicate that miR167 may interact with *TaMYC3* (Fig. 10). With an experiment under MeJA treatment (Fig. 6), it was speculated that *TaMYC*3 might be a core factor in JA-light crosstalk, which needs to be verified by further experiments.

## Conclusions

In this study, a comprehensive overview of the *MYC* gene family in wheat, including gene structures, phylogenetic relationships, and expression profiles, was provided. The roles of *TaMYCs* in response to abiotic stresses and light quality conversion were preliminary revealed. Moreover, our results provided insights into the crosstalk between MeJA signaling and light signaling in PTGMS wheat line BS366.

## Methods

### Plant materials, growth conditions, and sample collection

Wheat PTGMS line BS366 (winter wheat) were planted in the experimental fields in Beijing (China, N 39°54′, E 116°18′) and managed conventionally.

For tissue-specific expression analysis, wheat seedlings were grown until reaching the heading stage in experimental fields. Root, stem, leaf, petal, pistil, stamen, and glume of wheat were collected for tissue-specific expression analysis.

For abiotic stresses, two-week-old wheat seedlings were cultured in the thermostatic artificial climate chamber (CLC-BIV-M/CLC404-TV, MMM, Germany) (20 °C, 12 h day/12 h night cycle). For low temperatures stress, seedlings were placed in an incubator at 10 °C (12 h day/12 h night cycle). For salinity and drought stresses, seedlings were treated with 200 mmol/L NaCl solution and 20% PEG 6000 (− 0.5 MPa), respectively. For hormone treatments, seedlings were sprayed with 100 mmol/L ABA, 50 mmol/L IAA, or 100 mmol/L GA, with water as the control. Leaf tissue was collected from seedlings after 0, 4 and 12 h of stress treatment.

For effect of exogenous MeJA analysis, anthers of seedlings planted in experimental fields were treated with 0, 2 and 4 mmol/L MeJA solution at the heading stage. This experiment was carried out at the same time point within 5 days. Then collected at the anthers to investigate the effect of exogenous MeJA on the expression patterns of *MYC*s.

For effect of light quality conversation, four-leaf stage seedlings cultured in experimental fields were transferred into an artificial climate incubator with a 12-h photoperiod at a light intensity of 600 μmol/(m^2^*s) using fluorescent lamps. Night broken was performed at middle night (ZT = 18 h) using red (λ = 660 nm, 50 μmol/(m^2^*s)) and far-red light (λ = 731 nm, 40 μmol/(m^2^*s)) for ten days. Anthers were collected to determine the effect of conversion between red and far red lights on *TaMYCs* expression patterns. All samples were rapidly frozen in liquid nitrogen and stored at − 80 °C.

### Identification of the *MYC* gene family in wheat

The genome sequence data and the annotation information of wheat (Chinese spring) were obtained from the *Ensembl Plants* database (http://plant.ensembl.org/index.html). MYC protein data of *Arabidopsis thaliana* (At) and *Oryza sativa* (Os) were downloaded from TIAR (http://www.*Arabidopsis*.org/) and TIGR (http://www.jcvi.org/).The hidden Markov models (HMM) of MYC protein (Pfam accessions: PF14215 and PF00010) were downloaded from the Pfam database (http://pfam.xfam.org/) [40], and were used as queries to search for potential MYC proteins in the wheat protein data sets by using HMMER3.0 [41] with an E-value cutoff of 1.0E-05. Subsequently, the nucleotide and genomic sequences of each *TaMYC* gene copy was confirmed based on the database accession number of protein. In order to confirm conserved domains of MYC, candidate protein sequences were subjected to online domain analysis program NCBI–CDD (https://www.ncbi.nlm.nih.gov/cdd/), and SMART (http://smart.emblheidelberg.de/).

### Naming scheme of wheat *MYC* gene family

*MYC* gene copies isolated in this study were defined as *TaMYC*. The different copies of same *TaMYC* gene in A, B, and D sub-genomes were clustered based on the phylogenetic results. The order of each *TaMYC* gene was decided based on their locations on A, B, and D wheat chromosomes and the clustering relationship on the cladogram.

### Chromosomal locations and synteny analysis

Positional information of *TaMYC* genes were collected based on the genome annotation information. The synteny analysis of *TaMYC* genes was performed by using the whole genome synteny block data, which was available in the Plant Genome Duplication Database (http://chibba.agtec.uga.edu/duplication/) [42]. The visualization of chromosomal locations of *TaMYC* gene family was carried out by using Circos-0.69 (http://circos.ca/) [43].

### Physicochemical characteristics and *cis*-elements analysis

Protein sequence length, theoretical isoelectric point and molecular weight of TaMYC proteins were predicted by using the ExPASy online tool (http://www.expasy.org/). Subcellular localization were predicted by using the WoLEPSORT online tool (http://www.genscript.com/wolf-psort.html). To investigate putative *cis*–acting regulatory elements in the promoter regions of *TaMYC* genes, 1500 base pair genomic DNA sequences upstream of the initiation codon were retrieved and screened against the Plant CARE database (http://bioinformatics.psb.ugent.be/webtools/plantcare /html/) [44].

### Structural analysis of *TaMYC* genes and proteins

The full-length coding sequences (CDS) and genomic DNA sequences of the *TaMYC* genes were collected from wheat reference genome database (IWGSC; https://www.wheatgenome.org/). The structure analysis of *TaMYC* genes were performed via GSDS2.0 (http://gsds.cbi.pku.edu.cn/) [45]. Conserved motifs of TaMYC proteins were analyzed using the online program MEME [46].

### Multiple sequence alignment and phylogenetic tree construction

Multiple alignment of MYC protein sequences of wheat and other species (*O. sativa* and *A. thaliana*) was performed by using DNAMAN (ver. 6.0) with default parameters. An unrooted phylogenetic tree was constructed by using MEGA7.0 [47] with the neighbor–joining (NJ) method (1000 bootstrap trials, the Poisson model) based on the results of multiple alignment.

### Expression analysis of *TaMYC* genes

Total RNA extraction, first-strand cDNA synthesis and Quantitative real-time PCR (qRT-PCR) was performed according to Bai et al. (2018) [2]. The data were analyzed using the 2^-ΔΔCt^ method [48]. Each experiments were replicated three times. The primers used in this study were listed in Additional file 4: Table S1. Wheat actin gene (Genbank: AB181991) was used as the inner reference.

### miRNA targeting prediction of the *TaMYC* gene family

To investigate whether miRNA interacts with *TaMYC* genes, the known and studied *miRNA* sequences of wheat were collected from miRbase and subjected to online psRNATarget.

## Supplementary information

**Additional file 1: Figure S1.** Chromosomal localizations and syntenic relationships among *TaMYC* genes in *Triticum aestivum*, *T. urartu* and *Ae. Tauschii.* Lines in grey indicate tandem duplication. Lines in blue, green and orange indicate segmental duplication

**Additional file 2: Figure. S2.** Consensus sequence and logos of motifs from wheat MYC proteins

**Additional file 3: File S1.** Promoter sequences of *MYC* gene copies

**Additional file 4: Table S1.** The primers used in this study

**Additional file 5: Table S2**. Numbers of *cis*-regulatory elements in the upstream promoter regions of *TaMYC* genes

**Additional file 6: Table S3.** miRNA targeting prediction of *TaMYC* gene family

**Additional file 7: Table S4.** Segmental duplication of *TaMYC* genes

## Data Availability

The data sets supporting the results of this article are included within the article and its additional files.

## Abbreviations

ABA: Abscisic acid
GA: Gibberellin
IAA: Indole-3-acetic acid
MeJA: Methyl jasmonate
PTGMS: Photoperiod-Temperature sensitive Genic Male Sterile
