# Supplementary figures and images for: Genomic identification and characterization of MYC family genes in wheat (Triticum aestivum L.)
Source: BMC Genomics. 2019 Dec 30;20:1032. doi: 10.1186/s12864-019-6373-y (PMC6937671; doi:10.1186/s12864-019-6373-y)

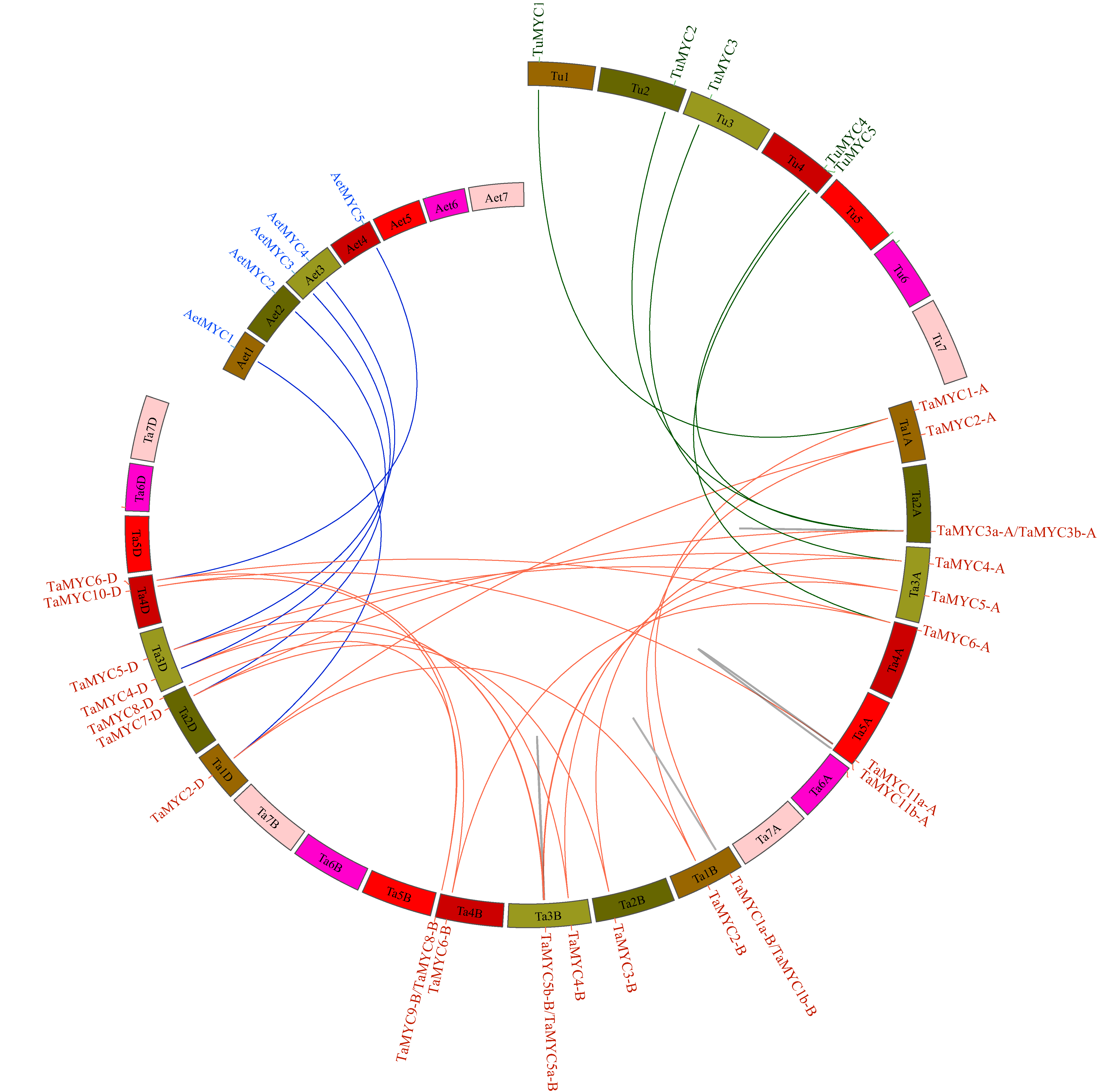

Supplement: Supplementary file 1 — Additional file 1: Figure S1. Chromosomal localizations and syntenic relationships among TaMYC genes in Triticum aestivum, T. urartu and Ae. Tauschii. Lines in grey indicate tandem duplication. Lines in blue, green and orange indicate segmental duplication [file 12864_2019_6373_MOESM1_ESM.tif]

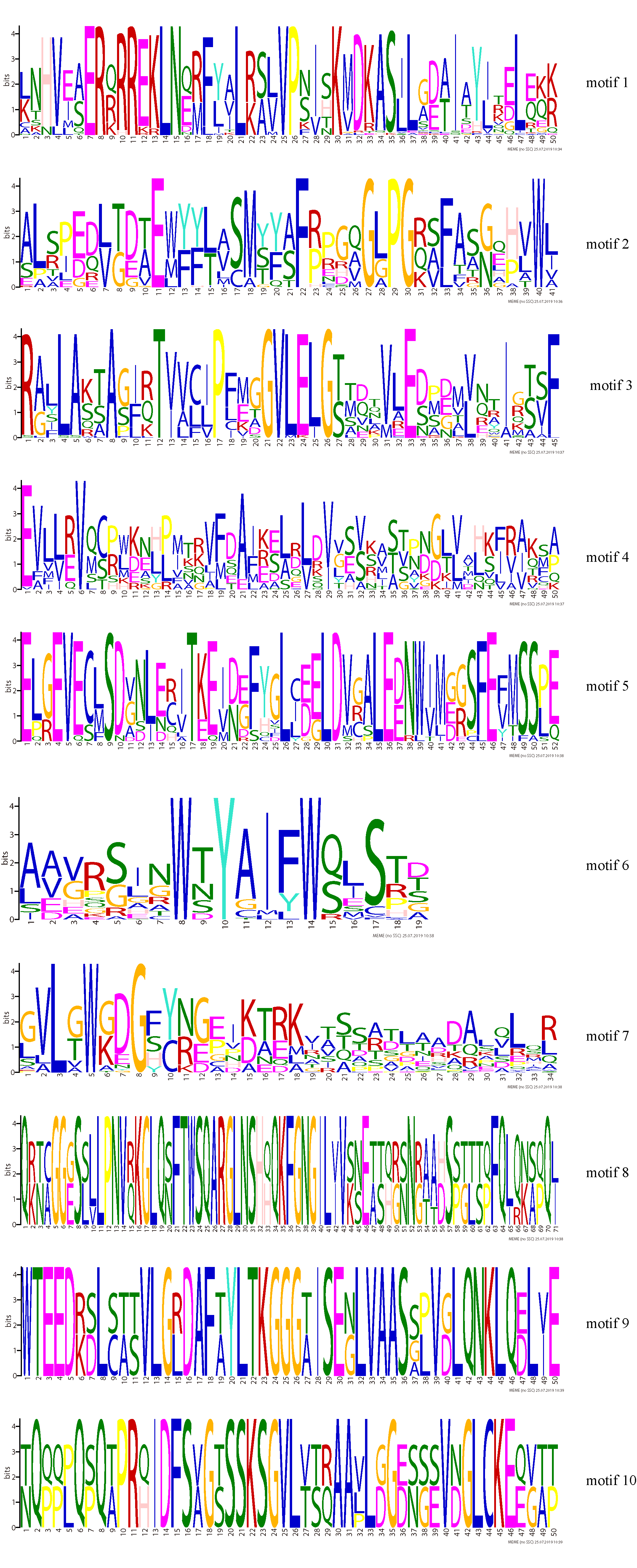

Supplement: Supplementary file 2 — Additional file 2: Figure. S2. Consensus sequence and logos of motifs from wheat MYC proteins [file 12864_2019_6373_MOESM2_ESM.tif]
