# Supplementary material for: Genomic identification and characterization of MYC family genes in wheat (Triticum aestivum L.)
Source: BMC Genomics. 2019 Dec 30;20:1032. doi: 10.1186/s12864-019-6373-y (PMC6937671; doi:10.1186/s12864-019-6373-y)
Supplement: Supplementary file 6 — Additional file 6: Table S3. miRNA targeting prediction of TaMYC gene family [file 12864_2019_6373_MOESM6_ESM.docx]

Table S3 miRNA Targeting Prediction of *TaMYC* Gene Family

| miRNA Acc. | Target Acc | miRNA start | miRNA end | Target start | Target end | miRNA_aligned_fragment | Target_aligned_fragment |
| --- | --- | --- | --- | --- | --- | --- | --- |
| *tae-miR1127b-3p* | *TaMYC5a-B* | 1 | 21 | 1739 | 1759 | ACAAGUAUUUCUGGACGGAGG | CCUCCGUUCCUAAAUACUUGU |
| *tae-miR9657a-3p* | *TaMYC7-D* | 1 | 21 | 1284 | 1304 | UGUGCUUCCUCGUCGAACGGU | ACCGGUCGACGGGGAGUCACA |
| *tae-miR9676-5p* | *TaMYC3-D* | 1 | 22 | 727 | 748 | UGGAUGUCAUCGUGGCCGUACA | ACCAUGGCCACGAUGAUGCCCG |
| *tae-miR9676-5p* | *TaMYC3a-A* | 1 | 22 | 727 | 748 | UGGAUGUCAUCGUGGCCGUACA | ACCAUGGCCAUGAUGAUACCCG |
| *tae-miR1138* | *TaMYC10-D* | 1 | 23 | 729 | 751 | GCUUAGAUGUGACAUCCUUAAAA | AGUUGAGGGGGUCACCUCUGAGC |
| *tae-miR167b* | *TaMYC3-B* | 1 | 21 | 965 | 985 | UGAAGCUGACAGCAUGAUCUA | CUGAUGGUGCUGUCACUUUUA |
| *tae-miR167b* | *TaMYC3-D* | 1 | 21 | 947 | 967 | UGAAGCUGACAGCAUGAUCUA | CUGAUGGUGCUGUCACUUUUA |
| *tae-miR5384-3p* | *TaMYC11a-A* | 1 | 21 | 533 | 553 | UGAGCGCGCCGCCGUCGAAUG | CCUUCAUGGGUGGCGUGCUCA |
| *tae-miR9773* | *TaMYC8-B* | 1 | 24 | 1107 | 1130 | UUUGUUUUUAUGUUAUUUUGUGAA | UCAAGAAAGUAACAUCAAGACACA |
| *tae-miR9773* | *TaMYC8-D* | 1 | 24 | 1107 | 1130 | UUUGUUUUUAUGUUAUUUUGUGAA | UCAAGAAAGUAACAUCAAGACACA |
| *tae-miR9773* | *TaMYC8-U* | 1 | 24 | 1107 | 1130 | UUUGUUUUUAUGUUAUUUUGUGAA | UCAAGAAAGUAACAUCAAGACACA |
| *tae-miR1128* | *TaMYC5a-B* | 1 | 21 | 1879 | 1900 | UACUACUCCCUCCG-UCCGAAA | UUUAGGAACGGAGGGAGUACUA |
| *tae-miR164* | *TaMYC6-D* | 1 | 21 | 478 | 498 | UGGAGAAGCAGGGCACGUGCA | UCCAUGUACUUUGCAUUUCCA |
| *tae-miR164* | *TaMYC6-A* | 1 | 21 | 478 | 498 | UGGAGAAGCAGGGCACGUGCA | UCCAUGUACUUUGCAUUUCCA |
| *tae-miR5384-3p* | *TaMYC11b-A* | 1 | 21 | 578 | 598 | UGAGCGCGCCGCCGUCGAAUG | CCUUCAUGGGUGGCGUGCUCG |
| *tae-miR9657c-3p* | *TaMYC7-D* | 1 | 21 | 1284 | 1304 | CGUGCUUCCUCGUCGAACGGU | ACCGGUCGACGGGGAGUCACA |
| *tae-miR9675-3p* | *TaMYC8-B* | 1 | 21 | 180 | 200 | UUUAUGAUCACUCUCGUUUUG | AAAGACGAGGAAGAUCAUAAG |
| *tae-miR9675-3p* | *TaMYC8-U* | 1 | 21 | 180 | 200 | UUUAUGAUCACUCUCGUUUUG | AAAGACGAGGAAGAUCAUAAG |
| *tae-miR9675-3p* | *TaMYC8-D* | 1 | 21 | 180 | 200 | UUUAUGAUCACUCUCGUUUUG | AAAGACGAGGAAGAUCAUAAG |
| *tae-miR9676-5p* | *TaMYC3b-A* | 1 | 22 | 727 | 748 | UGGAUGUCAUCGUGGCCGUACA | ACCAUGGCCACAAUGAUAACCG |
| *tae-miR9677a* | *TaMYC3-B* | 1 | 22 | 64 | 85 | UGGCCGUUGGUAGAGUAGGAGA | GAGCCCACCCUACCGCCGGCCG |
| *tae-miR9773* | *TaMYC11b-A* | 1 | 24 | 1155 | 1178 | UUUGUUUUUAUGUUAUUUUGUGAA | UCAAGAAAGUAACAUCAAGACGCA |
| *tae-miR9773* | *TaMYC11a-A* | 1 | 24 | 1110 | 1133 | UUUGUUUUUAUGUUAUUUUGUGAA | UCAAGAAAGUAACAUCAAGACGCA |
